# Genomic insight into the origins and evolution of symbiosis genes in *Phaseolus vulgaris* microsymbionts

**DOI:** 10.1186/s12864-020-6578-0

**Published:** 2020-02-27

**Authors:** Wenjun Tong, Xiangchen Li, Entao Wang, Ying Cao, Weimin Chen, Shiheng Tao, Gehong Wei

**Affiliations:** 1 State Key Laboratory of Crop Stress Biology for Arid Areas, Shaanxi Key Laboratory of Agricultural and Environmental Microbiology, College of Life Science, Northwest A&F University, Yangling, Shaanxi 712100 People’s Republic of China; 2 Bioinformatics Center, Northwest A&F University, Yangling, Shaanxi 712100 People’s Republic of China; 3 Departamento de Microbiología, Escuela Nacional de Ciencias Biológicas, Instituto Politécnico Nacional, 11340 México D.F, Mexico

**Keywords:** *Phaseolus vulgaris*, Horizontal gene transfer, Vertical gene transfer, Comparative genomics, Symbiosis genes

## Abstract

**Background:**

*Phaseolus vulgaris* (common bean) microsymbionts belonging to the bacterial genera *Rhizobium*, *Bradyrhizobium*, and *Ensifer* (*Sinorhizobium*) have been isolated across the globe. Individual symbiosis genes (e.g., *nodC*) of these rhizobia can be different within each genus and among distinct genera. Little information is available about the symbiotic structure of indigenous *Rhizobium* strains nodulating introduced bean plants or the emergence of a symbiotic ability to associate with bean plants in *Bradyrhizobium* and *Ensifer* strains. Here, we sequenced the genomes of 29 representative bean microsymbionts (21 *Rhizobium*, four *Ensifer*, and four *Bradyrhizobium*) and compared them with closely related reference strains to estimate the origins of symbiosis genes among these Chinese bean microsymbionts.

**Results:**

Comparative genomics demonstrated horizontal gene transfer exclusively at the plasmid level, leading to expanded diversity of bean-nodulating *Rhizobium* strains. Analysis of vertically transferred genes uncovered 191 (out of the 2654) single-copy core genes with phylogenies strictly consistent with the taxonomic status of bacterial species, but none were found on symbiosis plasmids. A common symbiotic region was wholly conserved within the *Rhizobium* genus yet different from those of the other two genera. A single strain of *Ensifer* and two *Bradyrhizobium* strains shared similar gene content with soybean microsymbionts in both chromosomes and symbiotic regions.

**Conclusions:**

The 19 native bean *Rhizobium* microsymbionts were assigned to four defined species and six putative novel species. The symbiosis genes of *R*. *phaseoli*, *R*. *sophoriradicis*, and *R. esperanzae* strains that originated from Mexican bean-nodulating strains were possibly introduced alongside bean seeds. *R*. *anhuiense* strains displayed distinct host ranges, indicating transition into bean microsymbionts. Among the six putative novel species exclusive to China, horizontal transfer of symbiosis genes suggested symbiosis with other indigenous legumes and loss of originally symbiotic regions or non-symbionts before the introduction of common bean into China. Genome data for *Ensifer* and *Bradyrhizobium* strains indicated symbiotic compatibility between microsymbionts of common bean and other hosts such as soybean.

## Background

Most legumes can establish mutualistic symbiosis with certain Alphaproteobacteria or Betaproteobacteria known as rhizobia [1]. Such symbiotic relationships have coevolved over millions of years and are fundamental to sustainable agriculture because they contribute approximately half of global terrestrial nitrogen nutrients [2]. *Phaseolus vulgaris* L. (common bean) is an important leguminous food crop cultivated worldwide in a broad range of cropping systems and environments. This species was domesticated from a wild-growing vine around 7000 years ago, in two primary centers of origin located in Mexico/Central America and the southern Andes (Ecuador, Peru, Chile, and Argentina) [3, 4]. Like many other legumes, common bean plants form efficient nitrogen-fixing nodules with diverse rhizobia [5–7]. To date, 18 *Rhizobium* species have been isolated from common bean root nodules. In addition, the symbiovar (sv.) mediterranense in *Ensifer* (*Sinorhizobium*) *meliloti* [8], *E*. *fredii* [9], and *E. americanum* [10] can nodulate common bean plants in alkaline-saline soils. Some of these rhizobia have been detected in both the centers of origin and other areas because they can be introduced with common bean seeds [11].

Common bean is believed to have been introduced into China directly from Latin America around 400 years ago [12], and China is now one of the world’s major producers of common bean. In previous studies, 371 rhizobial strains were isolated from root nodules of common bean plants grown in fields at 21 sample sites in four provinces in China [13, 14]. Approximately 89% of these isolates were *Rhizobium*, including six defined species and eight novel genospecies, while the remaining strains were *Bradyrhizobium* (2.4%) and *Ensifer* (8.4%) [13]. To date, several species such as *R*. *anhuiense* [15], *E. fredii* [14], and some genospecies from *Bradyrhizobium* [13] and *Ensifer* [14] have only been found in China. Some of these common bean-nodulating rhizobia were also found in association with other hosts, such as *Vicia faba* (fava bean) and *Trifolium* spp. (clover) in China [15]. How such an extraordinary variety of genotypes from three distinct genera (*Rhizobium*, *Bradyrhizobium*, and *Ensifer*) evolved into microsymbionts of common bean after its introduction into China is very interesting.

Concerning the location of symbiosis genes (on plasmids or chromosomes), *Rhizobium* species [16], *Mesorhizobium huakuii*, *M. amorphae* [17, 18], and *Ensifer* species [19] differ from *Bradyrhizobium* species [20, 21], *M. loti* [22], and *M. ciceri* [23]. Growing evidence indicates that horizontal gene transfer (HGT) of symbiosis plasmids/islands allowed diverse bacteria to engage in symbiosis with different leguminous host plants during the evolution of rhizobia [24, 25]. Hosts also play a role in HGT; for example, the roots of *Sesbania rostrata* secrete flavonoids that induce nodulation in the rhizobium-legume mutualistic symbiosis while enhancing the transfer of *Azorhizobium caulinodans* symbiosis islands [26]. However, it is unclear whether common bean enhances HGT in rhizobia during its adaptation to the introduced environment. Furthermore, common bean and *Glycine max* L. (soybean), both members of the Phaseoleae family, diverged 19 million years ago [27]. Soybean is a major leguminous crop originating in East Asia and it has been planted in China for over 5000 years. Different gene pools of soybean and its microsymbionts (mainly *Ensifer* and *Bradyrhizobium* strains) have been reported in various ecoregions of China [28, 29]. Therefore, the relationships of symbiosis genes in *Ensifer* and *Bradyrhizobium* strains that nodulate common bean and soybean in China are of interest.

Nodule-forming leguminous plants have been divided into three categories based on symbiotic specificities [30, 31]: (1) those stringently selected on both chromosomal and symbiosis gene backgrounds, such as *Medicago sativa* L. (alfalfa); (2) those stringently selected on symbiosis gene background only, such as common bean, *Robinia pseudoacacia* L. (black locust) [32], and *Aspalathus carnosa* [33]; (3) those nodulating with diverse rhizobia harboring different symbiosis genes, such as soybean [29] and *Sophora flavescens* [31]. Common bean belongs to the secondary category even though three genotypes of rhizobial symbiosis genes (sv. phaseoli, sv. tropici, and sv. mimosa) have been identified [15]. Approximately 20 genospecies in sv. phaseoli (including *R*. *etli* CFN42^T^ and *R. esperanzae* CNPSo668^T^) with different chromosomal backgrounds share *nodC* gene similarities of 97.3–100% [15]. In contrast, strains in sv. tropici and sv. mimosa harbor symbiotic genes different from those of sv. phaseoli, and these strains have a wide host range [15, 34, 35]. In these cases, symbiosis genes, typically those involved in nodulation (*nod*, *nol*, and *noe*) [36] and nitrogen fixation (*nif*, *fix*, and *fdx*) [37] in rhizobia, might have been transferred vertically in some species, but horizontally in others [38]. However, a plant introduced into a new environment could acquire indigenous rhizobia originally associated with a native legume species [39].

To clarify how different rhizobial species were recruited as symbionts of common bean in China, comparison of individual symbiosis genes, such as *nodC*, could provide insight into the phylogenetic relationships [40]. So far, more than 500 genes have been identified to be involved in rhizobium-legume symbiosis (e.g., *nod*, *nif*, *nol*, *fix*, *exo*, and *lps*) [41, 42]. Analysis of individual gene still lacks information on the genetic structure and interactions of symbiosis genes. Fortunately, genomics has revolutionized the way for estimation of the phylogenetic relationships among microbes, including the evolution of rhizobia [41]. In particular, comparison of whole genomes could contribute to our understanding about the relationships between rhizobia in the countries of origin and introduced regions.

Herein, we chose 25 representatives of common bean -nodulating rhizobia from China (19 *Rhizobium*, two *Ensifer*, and four *Bradyrhizobium*), and four from Mexico (two *Rhizobium* and two *Ensifer*), for comparative genomics analysis with reference strains. The genome analyses could shed light on different genetic associations while fully explaining the genetic structure of rhizobial strains. The goals of this study were (i) to estimate the origins of symbiosis genes among the common bean-nodulating rhizobial strains belonging to *Rhizobium*, *Ensifer*, and *Bradyrhizobium*, and (ii) to gain genomic insight into different symbiovars among the rhizobia investigated.

## Results

### Genomic features of core and accessory *Rhizobium* genomes

To analyze genomic features among strains in the *Rhizobium* genus occupying diverse niches, we used the 50 available *Rhizobium* genomes (Additional file 1: Table S1) to probe the evolution of the gene repertoire through pan-genome analysis. The pan-genome consisted of three parts, the core genome (shared by all strains), the dispensable genome (shared by some but not all strains), and the unique genome (unique to individual strains). The 50 *Rhizobium* strains were classified into 19 clusters or species (labeled R1 through R19) at the 95% average nucleotide identity (ANI) threshold for species delineation; this is consistent with the grouping results of digital DNA:DNA hybridization (dDDH) estimation and multilocus sequence analysis of housekeeping genes [15].

**Fig. 1:**
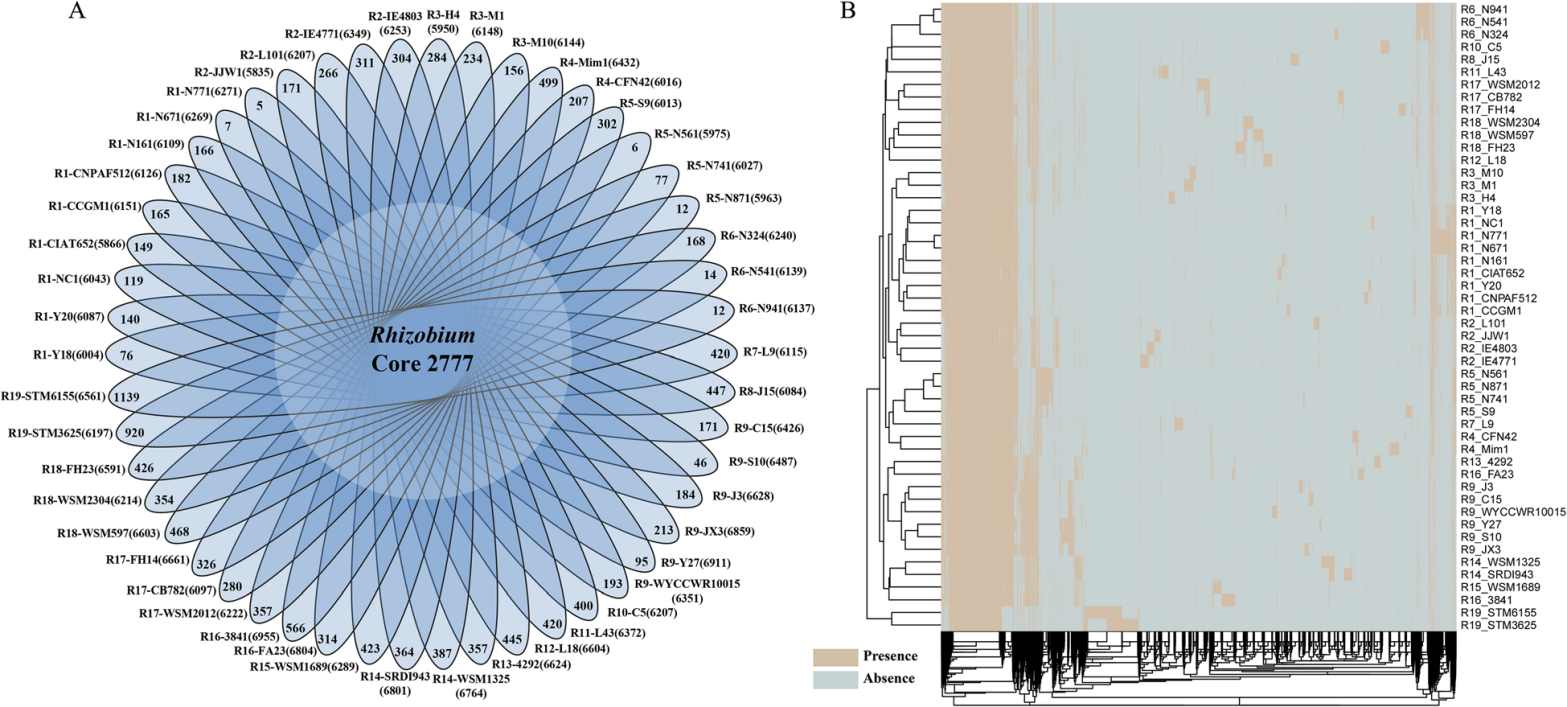
The pan-genome of 50 *Rhizobium* strains used in this study. **a** Flower plot showing the number of strain-specific genes (in petals) and core genes common to all *Rhizobium* strains (in the center). The name of each strain is preceded by the cluster number indicated in Additional file 1: Table S2. **b** Hierarchical clustering of *Rhizobium* genomes based on a heatmap of 30,767 genes in the pan-genome. The presence and absence of the 30,767 genes are indicated by bisque and azure, respectively

To better understand the pan-genome of *Rhizobium* strains, we clustered 315,181 coding sequences (CDSs) obtained from the 50 available genomes. This yielded a pan-genome containing 30,767 homologous gene families in the genus *Rhizobium*, with 2777 homologous genes in the core genome and 14,243 genes in the dispensable genome. The core genome represented 39.93 to 47.59% of the repertoire of protein-coding genes in each strain. Moreover, 13,747 genes belonging to the unique genome represented only one strain of *Rhizobium* (Fig. 1a). The number of strain-unique genes varied from five (R1-N771, with 6800 CDSs) to 1139 (R19-STM6155, with 6561 CDSs). It is noteworthy that all five unique genes in R1-N771 (and all six unique genes in R5-N561) encode hypothetical proteins. Hypothetical protein-coding genes accounted for more than 61% of unique genes in each strain.

Furthermore, we used hierarchical clustering to construct bifurcating trees and identified strains sharing similar gene content based on the presence and absence of 30,767 genes in the pan-genome across the 50 *Rhizobium* genomes. The hierarchical cluster derived from these data clearly distinguished strains of the same species from those of different species (Fig. 1b). The clustering results were well supported by the inter-species assignments based on the neighbor-joining species tree of 2110 concatenated single-copy core genes shared by 50 *Rhizobium* genomes (Additional file 2: Figure S1).

### Species and host trees

To comprehensively understand the evolution of common bean *Rhizobium* microsymbionts, we chose 29 representative strains (Additional file 1: Table S1) in 10 genospecies (clusters) which comprised more than two strains each. The representative strains were used for the analysis of vertically transferred genes that could reflect phylogenetic relationships among these strains, and horizontally transferred genes that may be related to host specificity.

A total of 2654 single-copy core genes were extracted from the 29 representative strains and phylogenetic trees were constructed to identify genes supporting the known phylogeny of rhizobia. The Shimodaira–Hasegawa test for the comparison between the phylogenetic tree for each of the 2654 core genes and the species tree uncovered 191 genes with consistent phylogenies (Additional file 1: Table S2). Of these 191 core genes, none were found on symbiosis plasmids, and only five were detected on accessory plasmids, as indicated in the complete genome of *R*. *etli* CFN42^T^. Specifically, one gene encodes a hypothetical protein on plasmid p42b, while the other four genes encode a probable transcriptional regulator protein in the IclR family, an oligopeptide ABC transporter substrate-binding protein, an XRE family transcriptional regulator protein, and an oligopeptide ABC transporter substrate-binding protein, respectively, on plasmid p42e in *R. etli* CFN42^T^. Most (186 out of the 191) species-related genes were located on chromosomes. With universal distribution and strictly vertical transfer among the 29 rhizobial genomes, 16 highly-conserved genes encode hypothetical proteins. These 16 genes may perform essential biological functions in the survival of rhizobial strains. In general, 65 genes were related to metabolism (e.g., *plsCX*, *fabAD*, *metCK*, *folC*, *mgsA*, *aglK*, *purF*, *serB*, *argCH*, and *dppB*), 24 genes were linked to translation and biogenesis (e.g., *murBC*, *exoN*, *hisS*, *rpsK*, *tlyA*, *tsf*, and *frr*), 18 genes were associated with transcription (e.g., *cspBG* and *nrdR*), and eight genes were involved in defense mechanisms and signal transduction mechanisms (e.g., *lolD*, *msbA*, *pleD*, and *dksA*; Additional file 1: Table S2).

Before identifying the core genes related to symbiotic specificity, we first carried out cross-nodulation tests with four legume species (*Trifolium pratense*, *Mimosa pudica*, *Phaseolus vulgaris*, and *Leucaena leucocephala*) to verify the symbiotic specificity. The rhizobium-legume symbiosis was highly specific, such that each rhizobial genus/species/strain could nodulate only a specific group of legume, and vice versa [43]. We then chose seven representative *Rhizobium* strains from clusters (species) containing the corresponding symbiovars. The results confirmed that all representative strains could nodulate their original host only (Additional file 2: Figure S2). Although R2-L101 possessed two types of *nod* gene clusters (partial *Phaseolus*-type and complete *Mimosa*-type; Additional file 2: Figure S3), this strain could not engage in symbiosis with *M. pudica* or *L. leucocephala*. Several core genes specifically related to the host of origin were found on symbiosis plasmids. Since symbiosis plasmids/islands can be transferred from an inoculant strain to a non-symbiotic strain, and since symbiotic regions are usually clustered in particular regions, we investigated the *nod*, *nif*, and *fix* gene clusters further. Twelve symbiosis genes (*nodABC*, *fixABC*, and *nifHDKENB*) were found to be related to the host of origin (Additional file 2: Figure S3). These genes represent diverse genomic organization and may act as the major determinants of symbiotic specificity.

### Recent HGT among *Rhizobium* strains

To investigate HGT events in the *Rhizobium* genus, we obtained the complete sequences of symbiosis plasmids from *R*. *acidisoli* FH23 for HGT analysis. The genome size of strain FH23 was 7,497,685 bp (135,772 bp larger than its draft genome), which comprised a chromosome (4.57 Mb) with a G + C content of 61.5% and four plasmids (0.67–0.85 Mb) with a G + C content of 58.4–61.1%. Most of the *nod*, *nif*, and *fix* symbiosis genes were clustered in a 0.1 Mb region on symbiosis plasmid pRapFH23a (Additional file 2: Figure S4).

To explore the effects of HGT among the 35 *Rhizobium* strains nodulating *P. vulgaris* (Additional file 1: Table S1), we employed a pairwise sequence conservation strategy known as RecentHGT [44]. A total of 447 species pairs among the 35 *Rhizobium* strains were found to share a significant number (> 50) of HGT genes (mean = 110, standard deviation = 39; Additional file 1: Table S3). The number of HGT genes shared between some species was considerably large; for example, there were 20 species pairs among R1, R2, R5, R9, R11, R12, and R13, sharing over 200 HGT genes. By contrast, only three species (R14, R15, and R19) shared few recent gene communication events. Moreover, the number of predicted HGT genes had no significant correlation (Spearman’s ρ = − 0.057, *p* = 0.58) with the ANI values between the 447 species pairs. The results indicate that phylogenetic distance was not a significant barrier for recent HGT events among *Rhizobium* species.

**Fig. 2:**
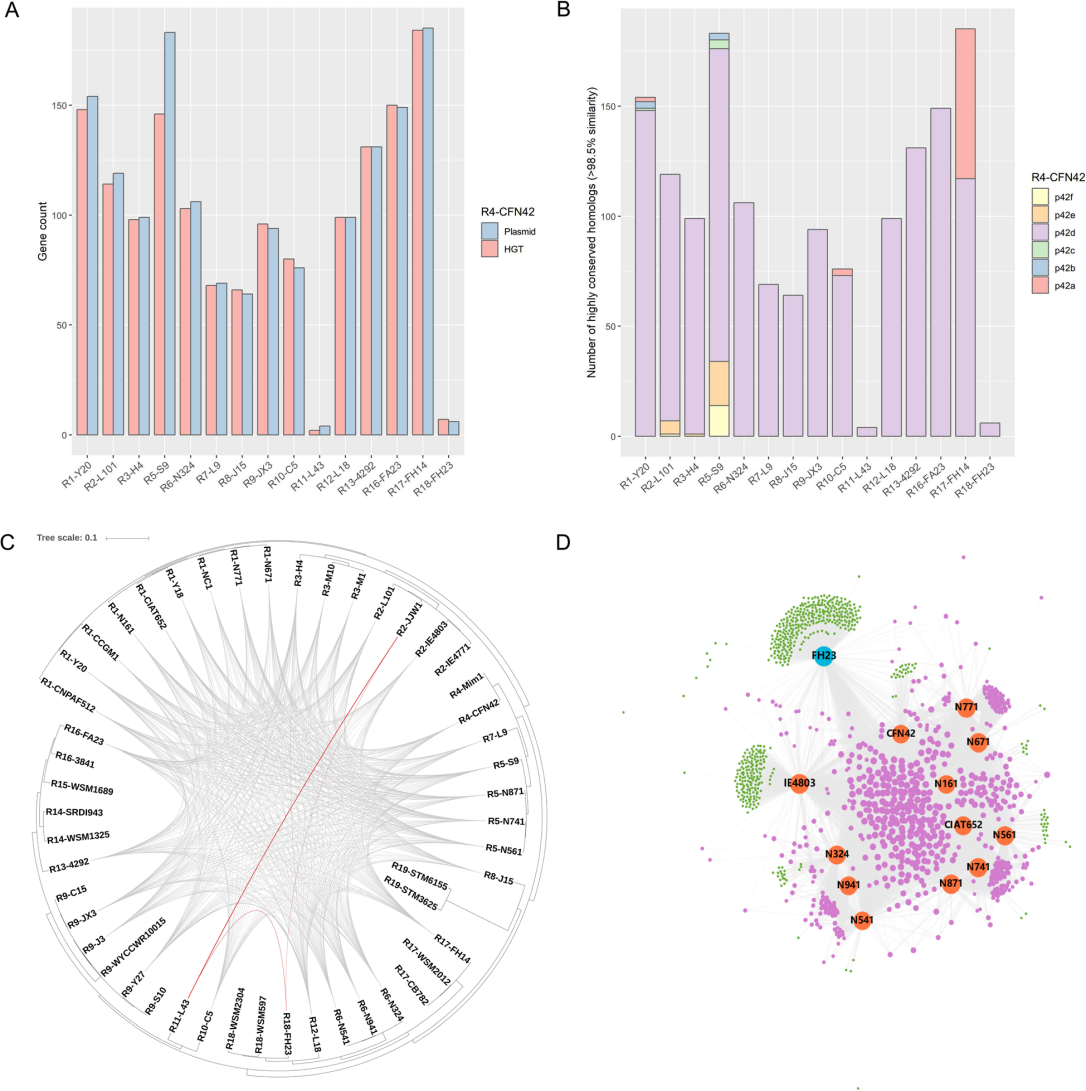
Extensive recent horizontal gene transfer (HGT) among the 50 *Rhizobium* strains. **a** Comparison of the predicted number of recent HGT genes and the number of highly conserved plasmid genes between the reference genome of R4-CFN42 (*R*. *etli*) and other sample strains. **b** Genomic locations of the predicted HGT genes between R4-CFN42 and other sample strains. **c** All HGT events in the tested *Rhizobium* strains. Connection thickness is scaled to the number of shared protein-coding sequences. The maximum likelihood tree is based on concatenated single-copy protein-coding gene alignments. **d** Bipartite network of 13 symbiosis plasmids from *Rhizobium* strains nodulating *P. vulgaris*. The two clusters of plasmids based on network clustering are represented as blue and orange nodes. Purple nodes represent gene families shared by at least two plasmids, while plasmid-specific gene families are indicated as green nodes. All edges connecting plasmids and gene families are denoted by gray lines

Based on the reference genome R4-CFN42, we found that the predicted number of HGT genes and the number of highly conserved homologs on plasmids were nearly identical among most of the HGT events between two species (Fig. 2a). In addition, most of the HGT genes were found on symbiosis plasmids, and on accessory plasmid p42a in strains R4 and R17 (R4-CFN4 and R17-FH14; Fig. 2b). Notably, some HGT genes were symbiosis genes (*nod*/*nif*/*fix*) and mobile elements, encoding plasmid proteins with key functions such as (1) replication (e.g., the *repABC* operon) and (2) conjugation (e.g., type IV secretion system and conjugative transfer relaxase). Moreover, we found that all inferred HGT events occurred within the *Rhizobium* species isolated from *P. vulgaris* root nodules only, and ANI values of some species pairs from different host plants were considerably smaller (Additional file 1: Table S3). Host-specific HGT events were consistent with host selection of symbiosis genes.

It is worth noting that two different HGT groups were identified based on the occurrence of HGT events among the 35 *Rhizobium* strains nodulating *P. vulgaris* (Fig. 2c), and there was a small group of only three strains (R2-JJW1, R11-L43, and R18-FH23). To explore the phylogenetic relationship of symbiosis plasmids in the two HGT groups, we constructed a bipartite ‘gene families-plasmids’ network of 13 symbiosis plasmids from the *Rhizobium* strains nodulating *P. vulgaris*, in which one partition represented plasmids and the other represented homologous gene families (Fig. 2d). We then performed a hierarchical clustering analysis and identified plasmid clusters at a 95% distance threshold. The network revealed that the symbiosis plasmid in R18-FH23 was distant from other symbiosis plasmids, sharing fewer homologous genes and containing more unique genes. Further, we analyzed functional enrichment of these unique genes using clusters of orthologous groups (COG) annotations (Additional file 2: Figure S5). Apart from the ‘general function prediction only’ category, unique genes were significantly enriched in ‘inorganic ion transport and metabolism’ and ‘amino acid transport and metabolism’ categories (Fisher’s exact test, *p* < 0.01). In summary, symbiosis plasmids in R2-JJW1, R11-L43, and R18-FH23 were substantially different from other symbiosis plasmids with regard to *P. vulgaris*-*Rhizobium* symbiosis.

### Relationships between microsymbionts of common bean and other hosts

**Fig. 3:**
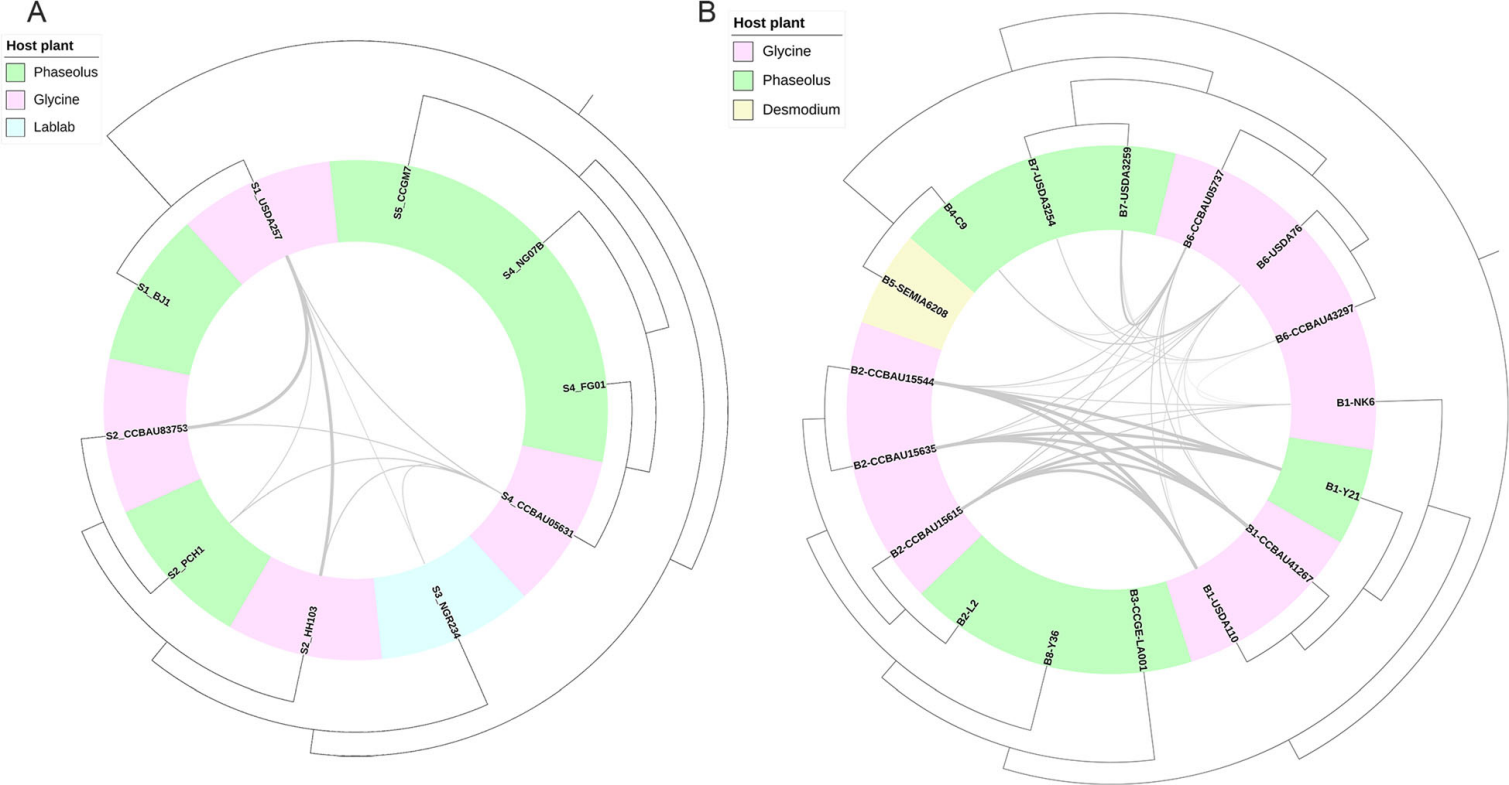
Genome comparison of *Ensifer* and *Bradyrhizobium* mostly isolated from common bean and soybean. **a** 10 *Ensifer* genomes. **b** 17 *Bradyrhizobium* genomes. Genome sequences were aligned using MAUVE v2.4.0, and the comparison was plotted using the R package ‘genoPlotR’

In the genome analysis of four *Ensifer* and four *Bradyrhizobium* microsymbionts of common bean, we obtained six and 13 related reference genomes from Genbank, respectively (Additional file 1: Table S2). At the 95% ANI threshold for species delineation, the 10 *Ensifer* strains and 17 *Bradyrhizobium* strains were divided into five and eight clusters, respectively, with an average aligned percentage of 85.88%. The result was consistent with their evolutionary relationships based on MAUVE alignments (Fig. 3) and dDDH values (Additional file 1: Table S4). Among the eight sequenced common bean microsymbionts, all four *Ensifer* strains and two *Bradyrhizobium* strains (Y21 and L2) displayed close relationships with soybean microsymbionts, while the other two *Bradyrhizobium* strains (C9 and Y36) exhibited unique genome content (Additional file 1: Table S4). Using the *RecentHGT* pipeline, we detected nine and 34 large-scale recent HGT events with more than 40 HGT genes among the *Ensifer* and *Bradyrhizobium* investigated, respectively. Unlike the *Rhizobium* strains, these common bean microsymbionts only shared HGT genes with the microsymbionts of other legume species, such as *Glycine max* and *Lablab purpureus* (Additional file 2: Figure S6; Additional file 1: Table S5).

We also analyzed 12 critical genes related to nodulation and nitrogen fixation (*nodABC*, *fixABC*, and *nifHDKENB*) in the eight common bean microsymbionts. Among the four *Ensifer* strains, *Ensifer* sp. III FG01 and NG07B, isolated from root nodules of common bean in Mexico, shared almost identical nodulation- and nitrogen fixation-related genes, and these genes differed from those in *Ensifer* sp. III CCBAU05631 isolated from nodules of soybean. Despite their highly similar genomic background, these three strains exhibited differences in their symbiosis gene content, which could reflect host characteristics. Thus, these two symbiovars may be indicative of *Ensifer* sp. III. Nodulation genes of FG01/NG07B were highly similar to those of *Acacia farnesiana* and *P. vulgaris* microsymbionts, and most similar to strains with different hosts (Additional file 1: Table S6). Similarly, *Ensifer* sp. I BJ1 possessed heterogeneous nodulation genes from soybean microsymbionts *Ensifer* sp. I USDA257; however, only *E*. *fredii* PCH1 shared high similarity with nodulation genes from soybean microsymbionts *E*. *fredii* HH103/CCBAU83753 and USDA257/NGR234/CCBAU05631, although they were assigned to other species (Additional file 1: Table S4). Cross-nodulation tests further verified that strains PCH1, CCBAU83753, and CCBAU05631 could effectively nodulate common bean and soybean.

Among the four *Bradyrhizobium* strains, *Bradyrhizobium* sp. I L2 possessed heterogeneous nodulation genes, implying diverse sources of origin. Nodulation gene extraction failed for strain Y36, consistent with its inability to nodulate with common bean or occasional formation of rod-like and whites nodules. *B*. *diazoefficiens* Y21 and *Bradyrhizobium* sp. III C9 possessed different nodulation genes. Specifically, nodulation genes of strain Y21 shared high similarity with those of soybean microsymbionts *B*. *diazoefficiens* USDA110^T^/NK6/CCBAU41267 and *Bradyrhizobium* sp. I CCBAU15615/CCBAU15635/CCBAU15544. Nodulation genes of strain C9 were highly similar to those of soybean microsymbionts *B. elkanii* CCBAU43297/CCBAU05737/USDA76. It appears that *Bradyrhizobium* microsymbionts of soybean possessed two sets of nodulation genes. Cross-nodulation tests revealed that both C9 and CCBAU43297 could effectively nodulate common bean and soybean while Y21, CCBAU41267, and CCBAU15615 formed white nodules with common bean and pink nodules with soybean. Strains Y21 and C9 isolated from nodules of common bean might be soybean microsymbionts. Their isolation from common bean indicates that this legume species can act as a promiscuous host.

## Discussion

In this study, we sequenced rhizobial genomes from 29 common bean microsymbionts in three distinct genera, *Rhizobium*, *Ensifer*, and *Bradyrhizobium*. The 29 rhizobial genomes were used to investigate the evolution of symbiotic genes among indigenous *Rhizobium* strains nodulating introduced bean plants, and to assess the emergence of an ability to engage in symbiotic relationships with bean plants in *Bradyrhizobium* and *Ensifer* strains.

We identified significant differences in both mean genome size and mean G + C content among rhizobial strains in the three distinct genera (Additional file 3), in agreement with previous work [41, 45]. Functional annotation based on the COG database indicated that larger genomes might be more inclined to include genes related to three particular functional categories, namely lipid transport and metabolism (I), secondary metabolite biosynthesis, transport and catabolism (Q), and defense mechanisms (V; Additional file 2: Figure S7). The trends of the first two categories (I and Q) are consistent with the genome analysis results of soybean microsymbionts (*Ensifer* and *Bradyrhizobium* strains) [41]. The result of the third category (V) supports an earlier study on gene content in the genomes of 115 prokaryotic species [46]. High correlations (|R| > 0.6, *P* < 0.001; Additional file 2: Figure S7) were obtained in approximately 30 pairs of gene functional categories; this result indicates that the functional categories complemented each other, as the identification of a series of metabolic pathways represents knowledge on the gene (molecular) interaction, reaction, and association networks [47].

Pan-genome analysis is an efficient tool for revealing the diversity and composition of the gene repertoire [48]. Herein, we used pan-genome analysis to characterize the gene repertoire of 50 available *Rhizobium* strains from different regions with diverse environmental conditions and host plants. The 50 *Rhizobium* strains were divided into 19 taxonomic clusters, with a shared core genome that represented less than half of the repertoire of protein-coding genes in each strain. Additionally, a high frequency of gene exchange with other taxa was evidenced by their large number of homologous gene in the dispensable and unique genomes. Of note, strains in the same cluster, despite isolated from diverse environments (countries or hosts), were more inclined to recruit lineage-specific shell genes under direct or indirect control through the speciation process, based on hierarchical clustering of the pan-genome. Concordance between pan-genome phylogenetic tree and core genome tree was also found for *Aeromonas hydrophila* [49], but not for multi-genus rhizobia [41]. In addition, an open pan-genome structure has been reported for rhizobial strains of *Bradyrhizobium*, *Ensifer* [41], and *Streptococcus* [50]. This pan-genome structure indicates that these rhizobial genera are able to acquire exogenous DNA and/or exchange genetic material in diverse environments [51].

Genetic material can be acquired by bacteria via gene transfer. In vertical gene transfer, genetic information is passed from parents to offspring by asexual splitting; in the case of HGT, a donor organism transfers genes to a recipient organism that is not a descendant mainly by transformation, conjugation, and transduction [52]. Here we constructed the species tree from single-copy core genes and analyzed vertically transferred genes. Comparison with the phylogenetic tree uncovered 191 genes with phylogenies consistent with the species tree. Apart from five core genes, most of the species-related genes were detected on chromosomes. This is because plasmids are more plastic, as demonstrated in the population genomics of sympatric *Rhizobium* species [16]. Although most core genes have various gene histories, the core-gene tree can still reflect the history of these rhizobia [53, 54]. Analysis of specific horizontally transferred genes related to the host of origin identified more genes than previously found [55]. However, a co-evolution process has been reported between *nod* genes and ribosomal/housekeeping genes for microsymbionts of *Acacia mearnsii*, indicating that microsymbionts of different host plants possess distinct evolutionary histories [56].

Based on comparison of the genomes of 50 *Rhizobium* strains, we assigned the 19 sequenced Chinese common bean microsymbionts to four defined species (*R*. *phaseoli*, *R*. *sophoriradicis*, *R*. *anhuiense*, and *R. esperanzae*) and six putative novel species [15]. Strains in *R*. *phaseoli*, *R*. *sophoriradicis*, and *R. esperanzae* were found to be closely related to Mexican strains, not only in terms of chromosomal background, but also in symbiosis genes. Such close phylogenetic relationships indicate that the three *Rhizobium* species share the same origin. *R*. *anhuiense* may be the dominant common bean microsymbiont in China, despite the reference strain of *R*. *anhuiense* WYCCWR10015 has been isolated from root nodules of *Trifolium repens* [57], and the *R*. *anhuiense* type strain CCBAU23252^T^ is from *Vicia faba* [58]. In the phylogenies of 12 symbiosis genes (Additional file 2: Figure S3), we found that the topologies of strains in *R*. *anhuiense* (R9) were related to their host of origin. Furthermore, three symbiovars (sv. viciae, sv. phaseoli, and sv. trifolii) have been identified in this species [15]. The symbiosis plasmids of these three symbiovars in *R*. *anhuiense* possess different *repABC* operons, suggesting their independent origins. It appears that *R*. *anhuiense* is more flexible in terms of acquiring symbiosis plasmids. Thus, common bean microsymbionts might be microsymbionts of other indigenous legumes before the introduction of common bean into China. In addition to *R*. *anhuiense*, another six putative novel species were exclusive to China, although they share many recently transferred symbiosis genes with *R*. *etli* CFN42^T^. Therefore, these strains might have transitioned from microsymbionts of other legumes or soil saprophytes to bean endosymbionts. Herein, we present strong evidence that indigenous *Rhizobium* strains likely acquired their common bean-specific symbiosis genes from other *Rhizobium* species that were introduced alongside bean crops.

The transfer of symbiosis genes mediated by symbiosis plasmids from *R*. *etli* to other species in introduced regions appears to be a common phenomenon [40]. Herein, we did not identify any strains assigned to *R. tropici*, which is thought to be indigenous to Brazil, and which has been successfully applied for bean inoculation in Brazil [59]. Nonetheless, we identified Chinese bean microsymbionts of three defined species sharing the same origin as Mexican strains, and large-scale HGT might have occurred in seven species in the *Rhizobium* genus. Despite the high plasticity of symbiosis plasmids, they appear to have been transferred in an interspecies manner within the *Rhizobium* genus only. Our results suggest that dispersion of key symbiosis genes is rare between rhizobial generaa; however, expansion within the same genus is frequent, which could explain the emergence of multi-symbiovars [1]. In addition, we found that *Ensifer* and *Bradyrhizobium* strains possess diverse symbiotic regions compared with *Rhizobium* strains. Comparative genomic analysis and cross-nodulation tests of *Ensifer* and *Bradyrhizobium* strains indicated symbiotic compatibility between soybean microsymbionts and common bean microsymbionts. Nodulation genes of *Ensifer* strains FG01/NG07B/BJ1 and *Bradyrhizobium* strain L2 are heterogeneous, as they are diverse when compared with similar strains interacting with a different host.

Rhizobia can live as saprophytes in soil or establish symbiotic relationships with legumes, and they can change their host characteristics through HGT of symbiosis plasmids [60]. However, little information is available on where HGT events have occurred. This raises an interesting question: were non-symbiont strains driven to obtained “new skill” in the soil by themselves or in the nodules by plants or by other factors? Here we found that symbiosis genes in the *Rhizobium* genus belong to a phylogenetically compact group, although common bean can be nodulated by rhizobia from distinct genera; this result indicates that *Rhizobium* species exhibit a restricted host range. Overall, our findings provide insight into various aspects of rhizobial evolution, but experiments are needed to clarify the frequency of HGT of symbiosis genes among *Rhizobium* lineages, and the effects of host plants on transfer of these genes. HGT of specific symbiosis genes within rhizobial genera is an important mechanism that allows legumes to form symbioses with rhizobia adapted to particular soils. It is also critical to understand the strategies taken by different legume species to select particular partners. Such knowledge will facilitate the application of nitrogen fixation mechanisms to non-leguminous crops and thereby contribute to sustainable agriculture.

## Conclusions

Among the 19 sequenced Chinese common bean microsymbionts, strains of *R*. *phaseoli*, *R*. *sophoriradicis*, and *R. esperanzae* may have been introduced together with bean seeds from Mexico. Strains of *R*. *anhuiense* and six putative novel *Rhizobium* species are exclusive to China, and they appear to have evolved into common bean microsymbionts via HGT events with Mexican rhizobia after being introduced into China. Common bean-nodulating *Ensifer* and *Bradyrhizobium* strains possess symbiotic regions distinct from those in *Rhizobium* strains, indicating symbiotic compatibility between microsymbionts of soybean and common bean. These findings provide insight into symbiotic and housekeeping genes during rhizobial evolution.

## Materials and methods

### Microsymbionts used in this study

We chose a total of 29 common bean microsymbionts (21 *Rhizobium*, four *Ensifer*, and four *Bradyrhizobium*), including 25 from China and four from Mexico. Other reference genomes used in the study included 29 *Rhizobium* strains (17 *Phaseolus vulgaris* microsymbionts, eight *Trifolium* spp. microsymbionts, three *Mimosa* spp. microsymbionts, and one *Pisum sativum* microsymbiont), six *Ensifer* strains (four soybean microsymbionts, one *Phaseolus vulgaris* microsymbiont, and one *Lablab purpureus* microsymbiont), and 13 *Bradyrhizobium* strains (nine soybean microsymbionts, three *Phaseolus* spp. microsymbionts, and one *Desmodium heterocarpon* microsymbiont; Additional file 1: Table S1).

### DNA extraction, genome sequencing, and sequence assembly

The draft genomes of 29 common bean microsymbionts were obtained as previously described [15] using the Illumina HiSeq platform at Novogene (Beijing, China). In addition, *R*. *acidisoli* FH23 was cultured in tryptone yeast broth and genomic DNA was extracted using a DNA extraction kit (Catalogue No. 9763, TaKaRa, Dalian, China) following the manufacture’s instructions. The complete genome of *R*. *acidisoli* FH23 was sequenced using the PacBio RS II platform at Novogene. Low-quality reads were filtered using SMRT Analysis v2.3.0 with default parameters [61], and the remaining reads were assembled to generate one contig without gaps. The genome sizes and G + C contents of rhizobial strains in different genera were compared by Wilcoxon rank-sum tests. Genome completeness and contamination were assessed by CheckM v1.0.11 [62]. The draft genomes of 29 common bean microsymbionts and the complete genome of *R*. *acidisoli* FH23 have been deposited in the GenBank database under project accession no. PRJNA403813.

### Gene prediction and functional annotation

A whole-genome BLAST search [63] (E-value ≤1e-5, minimal alignment length percentage ≥ 40%) was performed against the COG database [64], KEGG (Kyoto Encyclopedia of Genes and Genomes) [47], GO (Gene Ontology) [65], and NR (Non-Redundant) database. Automatic annotation was conducted on the RAST web server [66]. Correlations and *p*-values among gene functional categories and genome size were obtained using the corr.test function in the R package ‘psych’ and the obtained correlation matrix was visualized using the R package ‘corrplot’.

### Orthologous gene identification and pan-genome construction

To identify homologous gene families and construct the pan-genome of all strains, the ITEP toolkit was used for generation and curation of protein families [67]. Orthologous clusters were generated by the Markov Cluster algorithm [68], using an inflation value of 2.0 and a cutoff value of 0.4 as described previously [15]. Single-copy core genes were aligned with mafft v7.271 [69] and reverse-translated with PAL2NAL v14 [70]. Alignments were concatenated to infer maximum likelihood phylogenies with RAxML v8.2.4 under the GTR model with a gamma correction (GAMMA) for variable evolutionary rates [71]. The best maximum likelihood phylogenies were generated using autoMRE bootstrap convergence test. Presence and absence patterns in the pan-genome were statistically analyzed using an ad-hoc Python script, and the heatmap was generated using the R package ‘pheatmap’.

### Plant nodulation assays, reinfection tests, and cross-nodulation tests

Five leguminous plant species were selected for reinfection tests, namely *Trifolium pratense* (red clover), *Mimosa pudica* (mimosa), *P. vulgaris* (common bean), *Leucaena leucocephala* (white popinac), and *Glycine max* (soybean). More than 50 alternative strains to be selected for genome sequencing were subjected to reinfection test with common bean. In addition, cross-nodulation tests were performed using 15 representative strains, including seven sequenced *Rhizobium* strains (R2-L101, R2-JJW1, R4-CFN42, R9-Y27, R9-WYCCWR10015, R17-FH14, and R18-FH23), three *Ensifer* strains (PCH1, CCBAU 83753, and CCBAU 05631), and five *Bradyrhizobium* strains (C9, Y21, CCBAU 43297, CCBAU 41267, and CCBAU 15615).

Briefly, leguminous plant seeds were washed with sterilized distilled water two to four times, followed by 95% ethanol (v/v) for 1–3 min. Then the seeds were surface-sterilized with 0.2% HgCl_2_ for 3–5 min. After rinsing and imbibition, the seeds were germinated on 0.8% water-agar plates in the dark [72]. Once the true leaves emerged, one to four seeds (based on differences in seed size and plant size) were transferred to a customized high-density polyethylene edible fungus cultivation bag (28 cm long, 7.5 cm wide) containing 800 mL of sterile vermiculite and perlite (2:1, v/v). Each bag was inoculated with 1 mL of the 24 to 72 h culture of rhizobial strains, in triplicate. Plants were grown in a greenhouse (temperature = 28/20 °C, day/night) and watered with 300 mL Fahraeus nitrogen-free mineral solution [73]. Controls were subjected to the same conditions, without rhizobial inoculation.

### Recent HGT detection

The RecentHGT automatic pipeline [44] was implemented with 1137 species pairs (ANI < 95%) to detect recent HGT events between two rhizobial species at the genome level and simultaneously estimate the number of transferred genes. The protein-coding sequences of all homologous genes between each species pair were retrieved from the pan-genome and aligned by the Needle tool in the EMBOSS package. The sequence similarity threshold for the expectation-maximization algorithm was set to 98.5%, and the sequence similarity distribution was drawn by the ‘ggplot2’ package in R.

### Bipartite analysis of the symbiosis plasmids

Symbiosis plasmids were identified in 13 completely sequenced rhizobial strains nodulating *P. vulgaris* (R1-CIAT652, R1-N161, R1-N671, R1-N771, R2-IE4803, R4-CFN42, R6-N941, R5-N561, R5-N741, R5-N871, R6-N324, R6-N541 and R18-FH23). A bipartite ‘gene families-plasmids’ network was constructed based on the 13 proteomes using AccNet v1.2. Specifically, all homologous proteins sharing > 40% sequence similarity were clustered with kClust parameters -s = 1.73, −c = 0.8, and -e = 1e-10. The bipartite graph was hierarchically clustered with a practical similarity threshold of 85%. The resulting network files including nodes, edges, and clusters were then imported into Gephi v0.92 to visualize the bipartite graph [74], and the relative gene content of each plasmid was displayed by making the diameter of each node proportional to its degree. Visualization of the network was performed using Gephi’s built-in ForceAtlas2 algorithm with default parameters, except for the following: approximate speed = 1.0, scaling = 200, gravity = 1.0, and ‘prevent overlap’.

### Calculation of ANI and dDDH values, and comparison of genomes

All pairwise ANI values among the 10 *Ensifer* strains and 17 *Bradyrhizobium* strains were calculated using JspeciesWS with the MUMmer algorithm [75], and corresponding pairwise dDDH values were calculated using GGDC v2.1 with the recommended Formula 2 [76]. Multiple genome alignment was performed using MAUVE v2.4.0 [77] to identify regions of sequence homology and rearrangements between the reference genome and query genome sequences. Alignments were visualized using the R package ‘genoPlotR’ [78].

## Supplementary information

**Additional file 1: Table S1**. Genomes used in this study. **Table S2.** Core genes for which phylogenies are strictly consistent with the species tree. **Table S3.** Recent horizontal gene transfer (HGT) predictions among 50 *Rhizobium* strains. **Table S4.** Average nucleotide identity (ANI, %) and digital DNA:DNA hybridization (dDDH, %) values of 10 *Ensifer* strains and 17 *Bradyrhizobium* strains based on whole-genome alignments. **Table S5.** Recent HGT predictions among 10 *Ensifer* strains and 17 *Bradyrhizobium* strains. **Table S6.** Identities (%) of nodulation- and nitrogen fixation-related genes between sequenced strains and their most closely related strains.

**Additional file 2: Figure S1.** Neighbor-joining species tree based on the 2110 single-copy core genes shared by 50 rhizobial genomes used in this study. The name of each strain is preceded by the cluster number indicated in Additional file 1: Table S1. The 21 *Rhizobium* strains in bold font were determined in this study. **Figure S2**. Cross-nodulation tests on *Trifolium pretense*, *Mimosa pudica*, *Phaseolus vulgaris*, and *Leucaena leucocephala* with seven representative *Rhizobium* strains. * *n* = 3 cultivation bags per treatment. ** bold italic letters before strain names indicate the original host. P, *Phaseolus vulgaris*; T, *Trifolium pretense*. **Figure S3.** Core genes of 29 representative *Rhizobium* strains related to hosts. Neighbor-joining trees of 12 critical symbiosis genes were constructed using 1000 bootstrap replicates (only bootstrap support > 60% is shown). Bar = 5% sequence divergence. Symbiotic characteristics are indicated by different colored solid lines (host; red = *Phaseolus vulgaris*, green = *Mimosa* spp., blue = *Trifolium* spp.). **Figure S4.** Graphical circular map of the complete genome of *R*. *acidisoli* FH23. The outermost circle represents the coordinates of the genome sequence. Circles from outside to inside indicate protein-coding genes, genes BLAST searched against COG, KEGG, and GO databases, non-coding RNA, deviations in G + C content (green/red), and G-C skew (light green/pink). Outermost to innermost positions indicate genes in forward and reverse orientations. **Figure S5.** Distribution of homologous genes based on COG assignment of unique genes within the symbiosis plasmid of R18-FH23 (*R*. *acidisoli*). **Figure S6.** Prediction of recent HGT genes among strains isolated from different legumes. a *Ensifer* strains. b *Bradyrhizobium* strains. The width of links is proportional to the number of predicted recent HGT genes. The darker the link color, the larger the predicted magnitude of the recent HGT event. **Figure S7.** Correlogram showing correlations between genome size, protein-coding sequences, and COG assignments of the 29 rhizobial strains in three distinct genera. Positive correlations are indicated by cells with lower triangles or circles in upper triangles colored blue from lower left to upper right. Negative correlations are represented by red coloring from the upper left to the lower right. The darker and more saturated the color, the greater the magnitude of the correlation. Weak correlations (near zero) are almost colorless

**Additional file 3.** Comparison of genomic features among three distinct rhizobial genera.

## Data Availability

The draft genomes of 29 common bean microsymbionts and the complete genome of *R*. *acidisoli* FH23 have been deposited in the GenBank database under project accession no. PRJNA403813.

## Abbreviations

ANI: Average nucleotide identity
CDSs: Protein-coding sequences
dDDH: Digital DNA:DNA hybridization
HGT: Horizontal gene transfer
