# Supplementary material for: Genomic insight into the origins and evolution of symbiosis genes in Phaseolus vulgaris microsymbionts
Source: BMC Genomics. 2020 Feb 27;21:186. doi: 10.1186/s12864-020-6578-0 (PMC7047383; doi:10.1186/s12864-020-6578-0)
Supplement: Supplementary file 2 — Additional file 2: Figure S1. Neighbor-joining species tree based on the 2110 single-copy core genes shared by 50 rhizobial genomes used in this study. The name of each strain is preceded by the cluster number indicated in Additional file 1: Table S1. The 21 Rhizobium strains in bold font were determined in this study. Figure S2. Cross-nodulation tests on Trifolium pretense, Mimosa pudica, Phaseolus vulgaris, and Leucaena leucocephala with seven representative Rhizobium strains. * n = 3 cultivation bags per treatment. ** bold italic letters before strain names indicate the original host. P, Phaseolus vulgaris; T, Trifolium pretense. Figure S3. Core genes of 29 representative Rhizobium strains related to hosts. Neighbor-joining trees of 12 critical symbiosis genes were constructed using 1000 bootstrap replicates (only bootstrap support > 60% is shown). Bar = 5% sequence divergence. Symbiotic characteristics are indicated by different colored solid lines (host; red = Phaseolus vulgaris, green = Mimosa spp., blue = Trifolium spp.). Figure S4. Graphical circular map of the complete genome of R. acidisoli FH23. The outermost circle represents the coordinates of the genome sequence. Circles from outside to inside indicate protein-coding genes, genes BLAST searched against COG, KEGG, and GO databases, non-coding RNA, deviations in G + C content (green/red), and G-C skew (light green/pink). Outermost to innermost positions indicate genes in forward and reverse orientations. Figure S5. Distribution of homologous genes based on COG assignment of unique genes within the symbiosis plasmid of R18-FH23 (R. acidisoli). Figure S6. Prediction of recent HGT genes among strains isolated from different legumes. a Ensifer strains. b Bradyrhizobium strains. The width of links is proportional to the number of predicted recent HGT genes. The darker the link color, the larger the predicted magnitude of the recent HGT event. Figure S7. Correlogram showing correlations between genome size, pr [file 12864_2020_6578_MOESM2_ESM.zip › Figure S2.pdf]

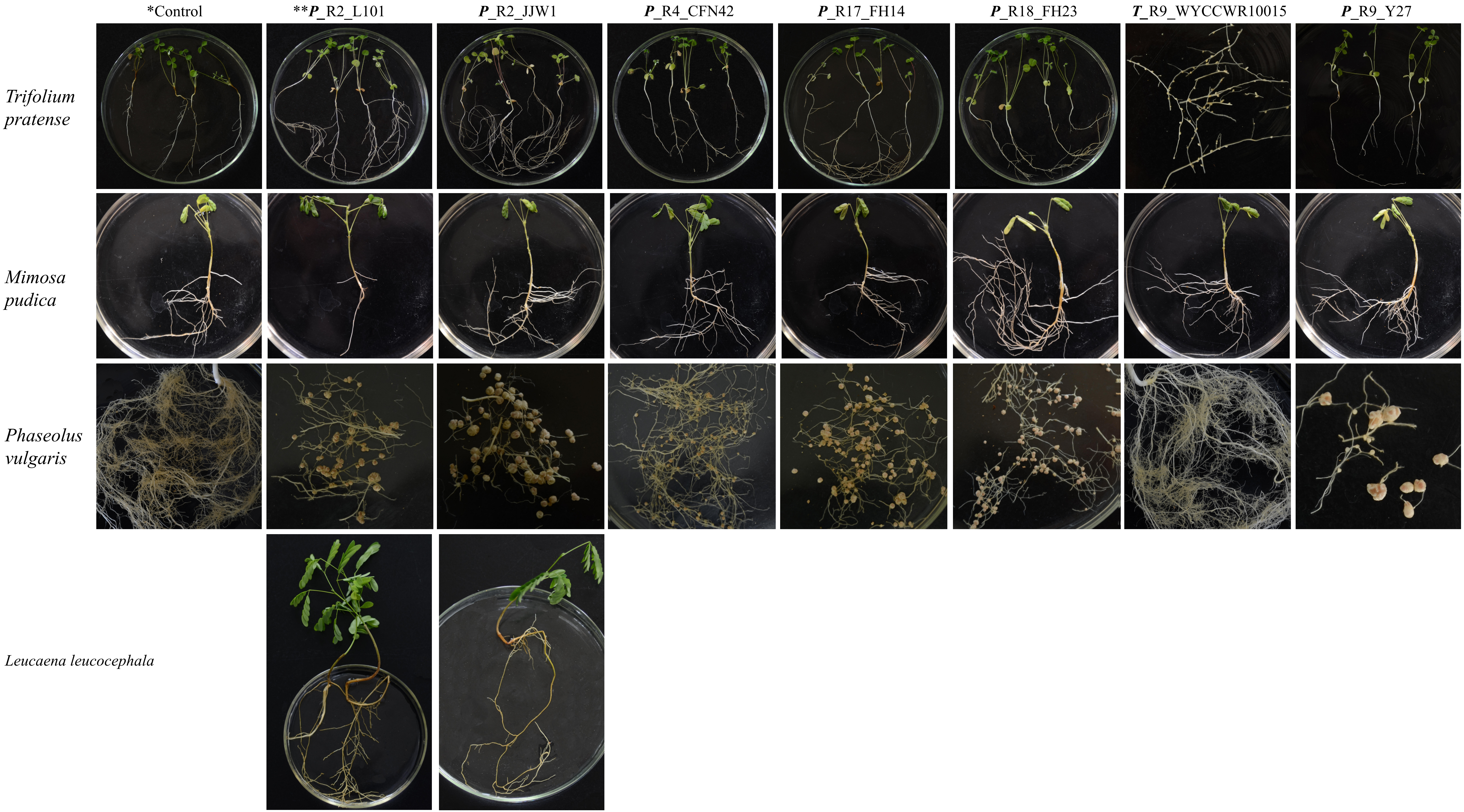
*Control
**P_R2_L101
P_R2_JJW1
P_R4_CFN42
P_R17_FH14
P_R18_FH23
T_R9_WYCCWR10015
P_R9_Y27
Trifolium pratense
Mimosa pudica
Phaseolus vulgaris
Leucaena leucocephala
