# Supplementary material for: Genomic insight into the origins and evolution of symbiosis genes in Phaseolus vulgaris microsymbionts
Source: BMC Genomics. 2020 Feb 27;21:186. doi: 10.1186/s12864-020-6578-0 (PMC7047383; doi:10.1186/s12864-020-6578-0)
Supplement: Supplementary file 3 — Additional file 3. Comparison of genomic features among three distinct rhizobial genera. [file 12864_2020_6578_MOESM3_ESM.docx]

**Results**

**Comparison of genomic features among three distinct rhizobial genera**

We sequenced the draft genomes of 29 rhizobial strains and reconfirmed their ability to nodulate common bean. There were 19 *Rhizobium* strains (representing 331 isolates with diverse BOX-PCR fingerprint patterns), four *Bradyrhizobium* strains (C9, L2, Y21, and Y36), and two *Ensifer* strains (BJ1 and PCH1) isolated from China, and two *Rhizobium* strains (*R*. *hidalgonense* FH14^T^ and *R*. *acidisoli* FH23) with two *Ensifer* strains (FG01 and NG07B) isolated from Mexico. Genomic features of the 29 newly-sequenced strains are summarized in Table 1. An average of 967 Mb of clean data (ranging from 659 Mb to 1,351 Mb) with deep sequence coverage (average 136×, ranging from 97-198×) was used to assemble genomes, resulting in an average scaffold of 102 (ranging from 52 to 158). Genome completeness was at least 99.7% for all 29 tested strains.

Genome sizes varied from 6.14 Mb (*Ensifer* sp. FG01) to 8.94 Mb (*Bradyrhizobium diazoefficiens* Y21), and the number of predicted protein-coding sequences (CDSs) ranged from 5,787 to 8,431 in corresponding strains. The genome size of *Rhizobium* strains was moderately flexible (6.35-7.67 Mb) with a G+C content around 61%. The mean genome size of *Bradyrhizobium* strains (8.37 Mb) was significantly greater than those of *Rhizobium* strains (6.94 Mb; *p* = 0.0034, Wilcoxon rank-sum test) and *Ensifer* strains (6.60 Mb; *p* = 0.0286). Furthermore, significantly differences (*p* <0.05) were identified in the mean G+C content among *Bradyrhizobium* strains (64.19%), *Ensifer* strains (62.15%), and *Rhizobium* strains (61.20%; Fig. 1). These 29 strains had the mean gene density of 86.77% with the lowest value (84.3%) observed in *Bradyrhizobium* sp. L2 (Table 1).


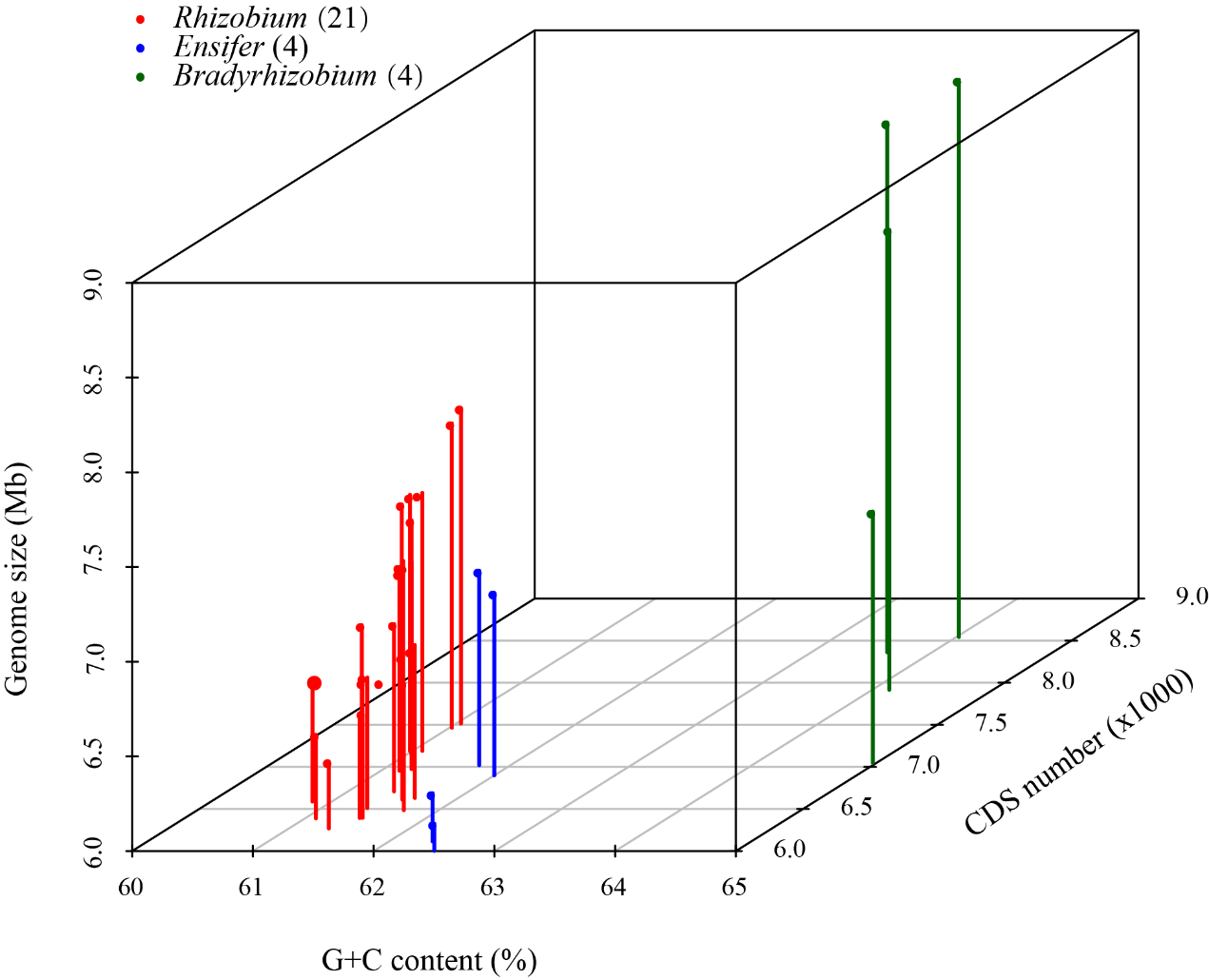


**Fig. 1** Three-dimensional plots of G+C content, genome size, and the number of predicted protein-coding sequences (CDSs) for the 29 sequenced *P*. *vulgaris* microsymbionts

**Table 1. Genomic features of the 29 newly-sequenced rhizobia strains isolated from common bean.**

|  | Clean data | | |  | General features of genomes | | | | | |  | Predicted CDSs | | |
| --- | --- | --- | --- | --- | --- | --- | --- | --- | --- | --- | --- | --- | --- | --- |
| Strain | | Length (Mb) | Depth |  | Scaffold number | N50 size (bp) | L50 (scaffold) | Completeness | Genome size (Mp) | G+C content (%) |  | Gene number | Coding density (%) | Assigned to COGs |
| R1-Y18 | | 703 | 108 |  | 64 | 385,108 | 6 | 99.94 | 6.534237 | 61.47 |  | 6,205 | 87.61 | 5,122 |
| R1-Y20 | | 830 | 117 |  | 148 | 149,660 | 15 | 99.94 | 6.689090 | 61.38 |  | 6,366 | 87.10 | 5,216 |
| R1-NC1 | | 860 | 119 |  | 119 | 205,115 | 11 | 99.94 | 6.675747 | 61.37 |  | 6,371 | 87.28 | 5,162 |
| R2-JJW1 | | 1,054 | 131 |  | 115 | 155,540 | 15 | 99.94 | 6.353547 | 61.33 |  | 6,146 | 87.23 | 5,044 |
| R2-L101 | | 989 | 123 |  | 110 | 182,046 | 13 | 99.89 | 6.883846 | 61.13 |  | 6,569 | 86.72 | 5,368 |
| R3-H4 | | 992 | 131 |  | 95 | 196,505 | 11 | 99.94 | 6.553177 | 61.45 |  | 6,271 | 86.67 | 5,141 |
| R3-M1 | | 1,029 | 127 |  | 115 | 183,505 | 13 | 99.94 | 6.784952 | 61.56 |  | 6,465 | 87.17 | 5,299 |
| R3-M10 | | 902 | 128 |  | 89 | 260,627 | 9 | 99.94 | 6.807137 | 61.64 |  | 6,462 | 87.46 | 5,346 |
| R5-S9 | | 869 | 140 |  | 83 | 287,606 | 8 | 99.85 | 6.440411 | 61.09 |  | 6,174 | 86.79 | 4,974 |
| R7-L9 | | 796 | 137 |  | 117 | 215,996 | 12 | 99.94 | 6.622380 | 60.84 |  | 6,394 | 86.73 | 5,201 |
| R8-J15 | | 1,002 | 137 |  | 116 | 132,538 | 15 | 99.94 | 6.670833 | 61.71 |  | 6,350 | 87.69 | 5,205 |
| R9-C15 | | 925 | 131 |  | 73 | 327,700 | 7 | 100.00 | 7.075381 | 61.15 |  | 6,748 | 87.90 | 5,646 |
| R9-S10 | | 859 | 139 |  | 72 | 285,812 | 9 | 100.00 | 7.094807 | 61.23 |  | 6,791 | 87.88 | 5,657 |
| R9-J3 | | 1,050 | 135 |  | 96 | 296,152 | 10 | 100.00 | 7.352769 | 60.98 |  | 7,000 | 87.40 | 5,739 |
| R9-JX3 | | 780 | 132 |  | 90 | 407,926 | 6 | 100.00 | 7.607295 | 61.02 |  | 7,227 | 87.24 | 5,928 |
| R9-Y27 | | 1,231 | 134 |  | 113 | 264,746 | 10 | 99.72 | 7.666520 | 61.04 |  | 7,324 | 87.20 | 5,958 |
| R10-C5 | | 1,024 | 149 |  | 96 | 230,095 | 9 | 99.94 | 6.883674 | 61.38 |  | 6,530 | 87.52 | 5,335 |
| R11-L43 | | 915 | 147 |  | 95 | 232,110 | 11 | 100.00 | 7.122309 | 61.22 |  | 6,763 | 86.81 | 5,479 |
| R12-L18 | | 910 | 145 |  | 108 | 242,699 | 11 | 99.96 | 7.236342 | 61.04 |  | 6,924 | 86.60 | 5,628 |
| R17-FH14 | | 845 | 145 |  | 136 | 181,699 | 13 | 100.00 | 7.268848 | 60.83 |  | 7,034 | 86.21 | 5,563 |
| R18-FH23 | | 1,048 | 167 |  | 122 | 213,669 | 10 | 99.96 | 7.361913 | 61.08 |  | 7,055 | 86.23 | 5,665 |
| S1-BJ1 | | 857 | 122 |  | 125 | 204,787 | 11 | 99.59 | 7.026577 | 61.74 |  | 6,792 | 85.88 | 5,334 |
| S2-PCH1 | | 956 | 137 |  | 133 | 178,622 | 12 | 100.00 | 6.964281 | 62.0 |  | 6,683 | 85.37 | 5,290 |
| S4-FG01 | | 659 | 107 |  | 70 | 336,222 | 7 | 99.96 | 6.144085 | 62.5 |  | 5,787 | 86.49 | 4,802 |
| S4-NG07B | | 1,239 | 198 |  | 52 | 649,830 | 3 | 99.96 | 6.254753 | 62.36 |  | 5,873 | 86.39 | 4,827 |
| B4-C9 | | 1,327 | 157 |  | 72 | 325,395 | 8 | 99.77 | 8.428461 | 64.14 |  | 7,868 | 85.92 | 6,030 |
| B2-L2 | | 1,351 | 154 |  | 158 | 251,037 | 10 | 99.89 | 8.797473 | 63.63 |  | 8,290 | 84.32 | 6,227 |
| B1-Y21 | | 865 | 97 |  | 126 | 367,887 | 7 | 99.98 | 8.941833 | 64.02 |  | 8,431 | 84.92 | 6,254 |
| B8-Y36 | | 1,180 | 161 |  | 54 | 217,818 | 9 | 99.98 | 7.327574 | 64.97 |  | 6,899 | 87.56 | 5,320 |

Genome characteristics of the 21 newly-sequenced *Rhizobium* strains have been published elsewhere [1].

N50 and L50 values are a set of scaffold lengths; N50 is the shortest sequence length at 50% of the genome, and L50 is the scaffold at 50% of the genome. CDS, coding Sequences; COGs, clusters of orthologous groups. The name of each strain was preceded by the cluster number indicated in Additional file 1: Table S1.

Reference

1. Tong W, Li X, Huo Y, Zhang L, Cao Y, Wang E, Chen W, Tao S, Wei G: **Genomic insight into the taxonomy of *Rhizobium* genospecies that nodulate *Phaseolus* *vulgaris***. *Systematic and applied microbiology* 2018.
